# Stimulation of hepatocarcinogenesis by neutrophils upon induction of oncogenic *kras* expression in transgenic zebrafish

**DOI:** 10.1016/j.jhep.2015.03.024

**Published:** 2015-08

**Authors:** Chuan Yan, Xiaojing Huo, Shu Wang, Yi Feng, Zhiyuan Gong

**Affiliations:** 1 Department of Biological Sciences, National University of Singapore, Singapore; 2 National University of Singapore Graduate School for Integrative Sciences and Engineering, National University of Singapore, Singapore; 3 MRC Centre for Inflammation Research, Queen’s Medical Research Institute, University of Edinburgh, 47 Little France Crescent, Edinburgh EH16 4TJ, UK

**Keywords:** HCC, hepatocellular carcinoma, FACS, fluorescence-activated cell sorting, Gcsfr, Granulocyte colony-stimulating factor receptor, TAN, tumor-associated neutrophil, NN, naïve neutrophil, RT-qPCR, reverse transcription-quantitative PCR, LPS, lipopolysaccharide, dpf, day post fertilization, H&E, hematoxylin and eosin, TUNEL, terminal deoxynucleotidyl transferase dUTP nick end labeling, Liver, Tumor-associated neutrophil (TAN), Inflammation, Tgf-β, Tumor initiation

## Abstract

**Background & Aims:**

Chronic inflammation is a major etiological factor for hepatocellular carcinoma (HCC), but how immune cells respond in the initiation of hepatocarcinogenesis remains uncharacterized. This study aims to investigate the response and roles of neutrophils in early hepatocarcinogenesis.

**Methods:**

By inducible expression of oncogenic *kras^V12^* in hepatocytes in transgenic zebrafish combined with live imaging of neutrophils in transparent larvae, the response of neutrophils to oncogenic liver was characterized and their roles investigated by pharmaceutical and genetic manipulations.

**Results:**

We found a rapid recruitment of neutrophils to the liver upon induction of *kras^V12^* expression. Pharmaceutical stimulation of neutrophils resulted in further increases of neutrophils in oncogenic livers, liver size and tumor severity, while inhibition of neutrophils caused decreases of liver-associated neutrophils and liver size. Time-lapse video indicated that neutrophils had a stagnant migratory pattern meandering along the tumor edge but became relatively stationary upon entering the *kras^V12^*-expressing liver. Both oncogenic hepatocytes and tumor-associated neutrophils (TANs) were isolated via fluorescence-activated cell sorting. Molecular analyses indicated a pro-inflammatory microenvironment, as marked by increased *tgfβ1a* expression in *kras^V12^*-expressing hepatocytes and a loss of anti-tumor activities in TANs. Depletion of Tgf-β significantly reduced the number of TANs and the size of oncogenic liver.

**Conclusions:**

An inflammatory cue from oncogenic hepatocytes upon induction of *kras^V12^* expression causes a rapid recruitment of neutrophils to oncogenic liver and the neutrophils play a promoting role in early hepatocarcinogenesis.

## Introduction

In the past decade, increasing evidence has indicated a dual role of neutrophils in a variety of tumors [1]. On one hand, activated neutrophils are capable of killing tumor cells through oxidative bursts [2] and secretion of anti-tumor cytokines such as TNF-α and IFNs [3]. On the other hand, in certain situations, neutrophils have also been found to promote tumor progression. Tumor-associated neutrophils (TANs) have long been observed to correlate to tumor progression in chronic colitis-associated carcinogenesis or gastric adenocarcinoma [4], [5], [6]. These alternatively behaved neutrophils are capable of releasing growth-stimulating signals, matrix-degrading proteases, and angiogenesis mediators [1], [7], favoring tumor progression. Recently, it has been reported the existence of subtypes N1 (anti-tumoral) and N2 (pro-tumoral) neutrophils; the neutrophil plasticity appears to be regulated by transforming growth factor-beta (Tgf-β), which is often found to be secreted by cancer cells [4]. Neutrophils are induced by Tgf-β to acquire an N2 phenotype, which differs from N1 neutrophils that require inhibition of Tgf-β and sufficient ifn-β [4], [8].

The influence between neutrophils and cancer cells are reciprocal. While tumor cells are capable of hyper-expressing pro-inflammatory molecules, mimicking the initial phase of wound healing [9], to attract neutrophils to the localized tumor microenvironment [10], the infiltrating neutrophils also have pro-angiogenic effects and promotes epithelial to mesenchymal transition during tumor progression [11], [12]. This integral relationship between immune cells and tumor cells is particularly evident in HCC, which is a typical inflammation-associated cancer since the primary etiological factors, hepatitis B and C viruses, create an unresolved, chronic inflammation of the liver [13]. To date, systemic therapy has not been effective in HCC patients [14], [15], although targeted therapy with a multi-kinase inhibitor, sorefenib, has limited efficacy in several clinical trials [16], [17]. Thus, immune-based therapy could be a new promising approach for HCC patients. The study of the interaction between HCC and neutrophils should provide much needed insights into the development of such a therapeutic approach.

Recently we have generated several inducible HCC models by transgenic expression of an oncogene in zebrafish hepatocytes [18], [19], [20], [21]. A major advantage of these inducible models is the temporal control of cancer initiation to provide an excellent opportunity to characterize tumor initiation events, hitherto lacking in human clinical studies and other non-inducible tumor models. Furthermore, the transparency of zebrafish larvae allows us to monitor early *in vivo* hepatocarcinogenesis and progression, thus providing a plethora of opportunities to investigate the initiation events in hepatocarcinogenesis and the roles of various cancer hallmark factors in the process. In particular, we recently observed a prominent immune response in HCC progression and regression in one of our zebrafish HCC models based on RNA-Seq analyses [22]. In the present study, the interaction of neutrophils and oncogenic hepatocytes in hepatocarcinogenesis was investigated. We observed a rapid recruitment of neutrophils into oncogenic liver, which led to accelerated tumor progression. Molecular analyses of fluorescence-activated cell sorting (FACS)-isolated hepatocytes and TANs indicated changes of several important molecular pathways, including promotion of a pro-inflammatory microenvironment in oncogenic hepatocytes and decreases of anti-tumor in TANs. Thus, our data suggest a promoting role of neutrophils in early hepatocarcinogenesis.

## Materials and methods

### Zebrafish husbandry

Zebrafish were maintained in compliance with Institutional Animal Care and Use Committee guidelines, National University of Singapore. Four transgenic lines, *Tg(fabp10:rtTA2s-M2; TRE2:EGFP-kras^G12V^)* in a Tet-On system for inducible liver-specific expression of oncogenic *kras^G12V^* [18], *Tg(lyz:DsRed*2*)*^nz50^ with DsRed-labeled neutrophils under the lysozyme C (*lyz*) promoter [23], *Tg(fabp10a:DsRed; ela3l:GFP)*^gz15^ with DsRed-labeled liver and GFP-labeled exocrine pancreas [24], *Tg(mpeg*1*:GFP)*^gl22^, with GFP-labeled macrophages under the *mpeg*1 promoter [25], were used and referred to as *kras*, *lyz*, *fabp10*, and *mpeg*, respectively, in the present report.

### Chemical treatment

20 μg/ml doxycycline (Sigma, D9891) was added from 3 days post-fertilization (dpf) to 8 dpf to induce *kras^G12V^-EGFP* expression. Lipopolysaccharides (LPS) (Sigma, L4391), FPR-A14 (Tocris, 2826), PR-39 (Tocris, 1947) and SB431542 (Tocris, 1614) were first dissolved in dimethyl sulfoxide as stocks and used for larva exposure at 5 ng/ml, 2.5 μM, 50 nM and 2.5 μM respectively from 4 to 8 dpf. The dosages were selected based on the highest all-survival concentrations.

### Statistical analysis

Statistical significance between two groups was evaluated by two-tailed unpaired Student *t* test using inStat version 5.0 for Windows (GraphPad, San Diego, CA). Statistical data are presented as mean value ± standard error of mean (SEM). Throughout the text, figures, and figure legends, the following terminology is used to denote statistical significance: ^*^p <0.05, ^**^p <0.01, ^***^p <0.001.

### Other methods

Other methods are described in Supplementary Materials and methods, including morpholino knockdown and Tgf-β depletion; photography and image analysis; isolation of hepatocytes and neutrophils by FACS; RNA extraction, cDNA amplification and RT-qPCR (reverse transcription-quantitative PCR); and histological and cytological analyses.

## Results

### Rapid recruitment of neutrophils into kras^V12^-expressing livers

**Fig 1:**
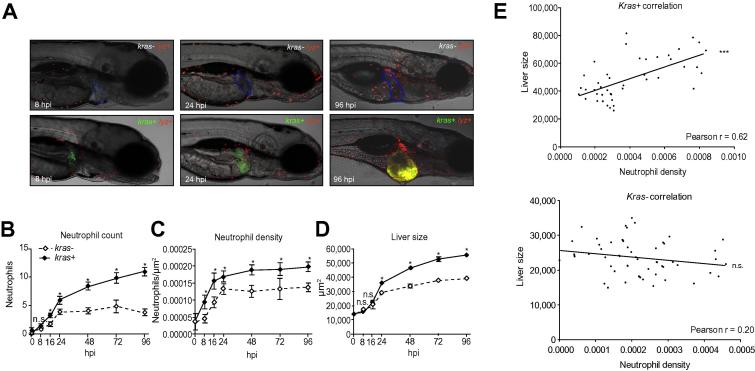
**Recruitment of neutrophils to oncogenic liver.** (A) Representative images of *kras−/lyz+* and *kras+/lyz+* larvae after 8, 24, and 96 hours of doxycycline induction starting from 3 dpf. The livers are outlined in blue dash lines in the upper images of *kras−/lyz+* larvae and marked by GFP expression in the lower images of *kras+/lyz+* larvae. (B–D) Time course of neutrophil count (B), neutrophil density (C) and liver size (D) following induction of oncogenic *kras^V12^* expression in hepatocytes (n >15 from each group). Neutrophils were counted in the liver area and normalized against the liver size for liver density. Liver size was measured based on 2D images. (E) Correlation of liver size and neutrophil density in the liver. The measurements were based on 8 dpf larvae after 5 days of doxycycline induction. A positive correlation was observed only in the *kras+* transgenic larvae (top) but not in the *kras−* control group (bottom). Statistical significance: ^*^p <0.05, ^**^p <0.01, ^***^p <0.001.

To visualize the inflammation response, hemizygous *kras+* transgenic fish were crossed with *lyz+* homozygous fish for production of *kras+* and *kras−* offspring in the *lyz*+ background, DsRed expressing neutrophils were monitored up to 96 hpi (hour post-induction with doxycycline). As shown in Fig. 1A and quantified in Fig. 1B–D, the total counts of neutrophils in the liver region was noticeably increased from as early as 8 hpi and became statistically significant from 16 hpi (Fig. 1B). These neutrophils within the vicinity of the liver, considered as TANs, were normalized against the liver size as neutrophil density. As shown in Fig. 1C, a significant increase in neutrophil density was observed from 8 hpi; thus, neutrophils were actively recruited to the site of tumor initiation within 8 hours of oncogene activation. In contrast, the increase of liver size became apparent only from 24 hpi (Fig. 1D), indicating that neutrophil recruitment preceded liver enlargement. To further evaluate the contribution of neutrophils to the increased liver size in *kras+* larvae, neutrophil density and liver size were plotted for each individual *kras+* larva and we observed a strong positive correlation (Pearson’s coefficient, 0.62); in contrast, such a correlation was not present in *kras−* siblings (Pearson’s coefficient, 0.20) (Fig. 1E).

### Acceleration of hepatocarcinogenesis by stimulation of general immune response and neutrophils

**Fig. 2:**
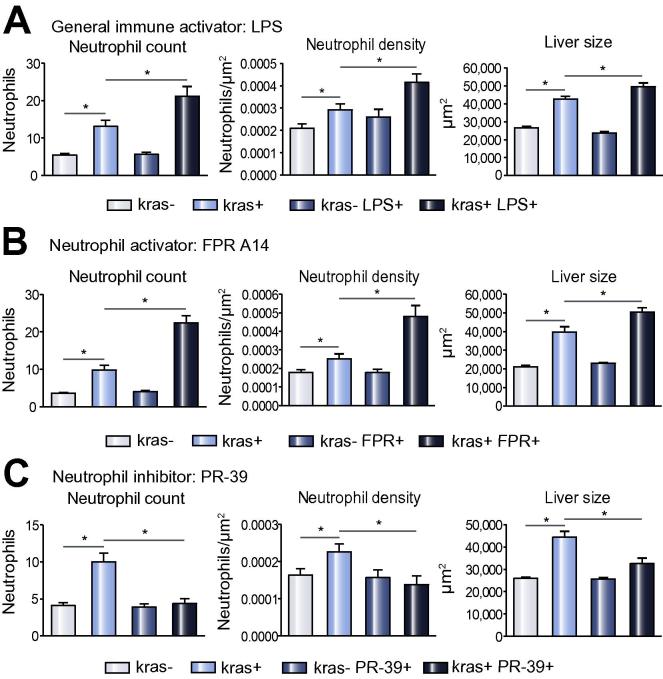
**Effect of infiltrated neutrophils on liver size.** (A–C) Neutrophil counts (left) and density (middle) in the liver area and liver size (right) in response to treatments of LSP (A), FPR A14 (B) or PR39 (C). Both *kras−/lyz+* and *kras+/lyz+* groups were similarly exposed to doxycycline from 3 dpf and neutrophils mediators were added from 4 dpf. Neutrophils and liver sizes were determined at 8 dpf (n >15 from each group). Statistical significance: ^*^p <0.05.

To further demonstrate the role of inflammatory cells in initiation and progression of hapatocarcinogenesis, we first tested a general inflammatory stimulator, LPS, which has been demonstrated to stimulate the immune system in zebrafish larvae [26]. 5 ng/ml of LPS was used to treat zebrafish larvae from 4 dpf to 8 dpf. *kras+* larvae exposed to both LPS and doxycycline showed significant increases of both neutrophil count and density in the liver as compared to *kras+* larvae exposed to doxycycline alone and all *kras−* groups. Interestingly, there was also a further enlargement of liver size with the increased neutrophils (Fig. 2A). To investigate if the accelerated liver enlargement was indeed associated with increased neutrophil infiltration, FPR-A14, which is a formyl peptide receptor agonist and has been reported to potently activate neutrophils specifically *in vitro* [27], was used to challenge the *kras+* larvae from 4 dpf to 8 dpf. Liver neutrophil count and density in FPR-A14 and doxycycline double-treated *kras+* larvae were also significantly higher than those of their *kras+* sibling treated with only doxycycline and of all *kras−* groups (Fig. 2B), similar to that observed following LPS treatment. A further liver enlargement was also observed from these double-treated *kras+* larvae. To further demonstrate the effect of neutrophils, *kras+* transgenic larvae were also challenged with a neutrophil inhibitor, PR-39, a proline-rich anti-bacteria peptide that inhibits NADPH oxidase activity in neutrophils [28]. As shown in Fig. 2C, liver neutrophil count and density as well as liver size in *kras+* larvae exposed to PR-39 and doxycycline were all decreased as compared to *kras+* sibling controls treated with doxycycline alone and all *kras−* groups. Thus, there was a good correlation between numbers of infiltrated neutrophils and the size of oncogenic liver, suggesting an *in vivo* promoting role of neutrophils in early hepatocarcinogenesis.

### Inhibition of neutrophil differentiation defers HCC progression

**Fig. 3:**
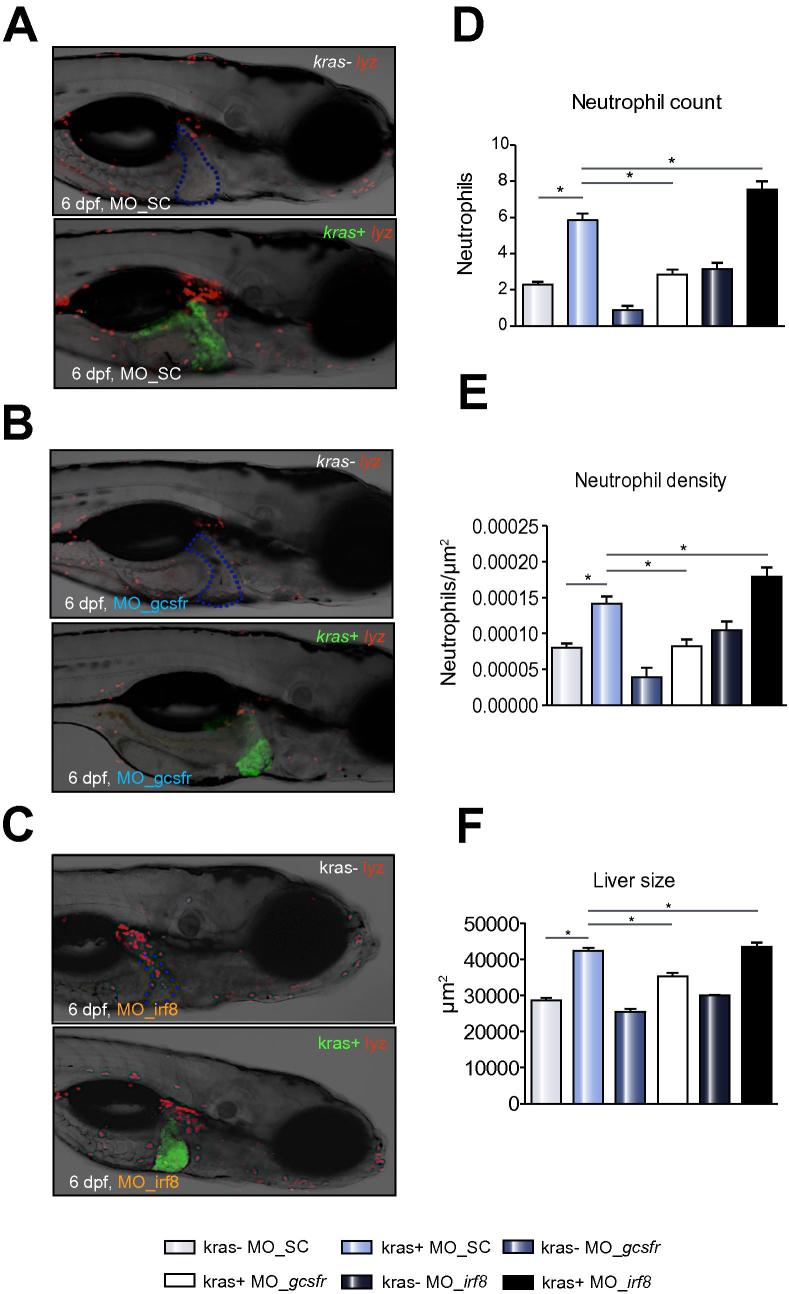
**Effect of morpholino suppression of neutrophil differentiation and morpholino-mediated expansion of neutrophil population on liver size.** (A–C) Representative images of 6 dpf *kras−/lyz+* and *kras+/lyz+* larvae injected with different morpholinos: Mo-SC (A), Mo-*gcsfr* (B) or Mo-*irf8* (C). (D–F) Neutrophil counts (D) and density (E) in the liver area and liver size (F) after morpholino injection. n >15 in each group. Statistical significant: ^*^p <0.05.

To further validate the effect of neutrophils on tumor growth, differentiation of myeloid derived precursor cells into neutrophils was blocked via morpholino knockdown of the *gcsfr* gene (MO_*gcsfr*). MO*_gcsfr* or control morpholino (MO_SC) were injected into *lyz+* embryos at one-cell stage and injected embryos were monitored for DsRed+ neutrophils. As shown in Supplementary Fig. 1A and C, by 6 dpf, there was an overall decrease of circulating neutrophils in *MO_gscfr* injected larvae, compared to those in *lyz+* larvae injected with MO_SC, consistent with the previous report that used the same set of morpholinos [29]. *kras+/lyz+* and *kras−/lyz+* zebrafish embryos were then injected with *MO_gscfr* and analysed at 6 dpf after doxycycline induction from 3 dpf. Similar to the observations with the PR-39 inhibitor, a significant decrease of neutrophil counts and density in the liver (Fig. 3D and E) as well as liver size (Fig. 3F) was observed, further confirming a promoting role of neutrophils in the initial stage of hepatocarcinogenesis.

To evaluate the role of macrophages in our model, depletion of macrophages was carried out by morpholino knockdown of the *irf8* gene [25]. As shown in Supplementary Fig. 1B and C, when *mpeg+* embryos were injected with MO_*irf8*, GFP expressing macrophages were greatly reduced; meanwhile, there was a compensating increase of neutrophils, consistent with a previous report that knockdown of *irf8* causes the common progenitor cells to differentiate to neutrophils [25]. When MO_*irf8* was injected into *kras+/lyz+* embryos, we observed a significant increase of neutrophil counts and density in the liver (Fig. 3C–E) with a corresponding increase of liver size (Fig. 3F). While the depletion of macrophages may not be conclusive for establishing their role in early hepatocarcinogenesis, it did demonstrate that neutrophils alone retained tumor prompting activity.

### Stagnant migratory pattern of tumor infiltrate neutrophils

**Fig. 4:**
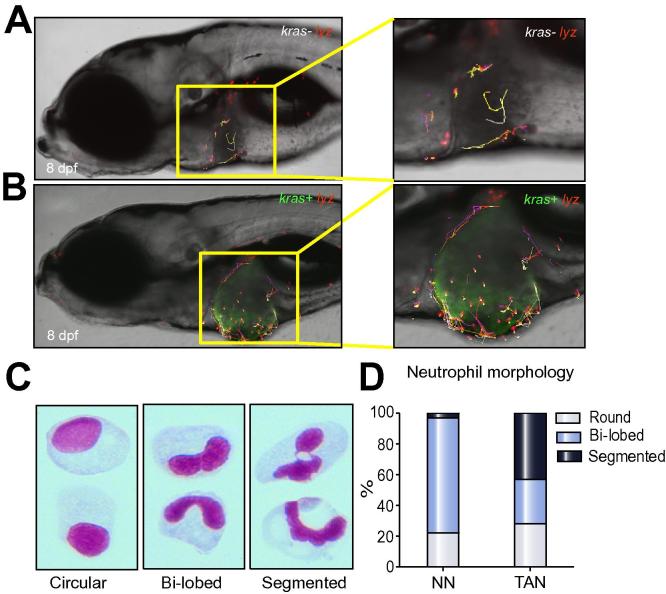
**Differential behaviors of NNs and TANs.** (A–B) Tracking of neutrophil movement within and surrounding the liver area in a *kras−/lyz+* larva (A) and a *kras+/lyz+* larva (B) respectively. Selected neutrophils were tracked based on one-hour time-lapse videos on 8 dpf larvae in Supplementary Videos 1 and 2. (C) Representative images of three distinct nuclear patterns after Giemsa staining: circular, bi-lobed and segmented nuclei. (D) Percentages of each nuclear pattern in NNs and TANs.

Neutrophil migratory pattern was also examined using confocal time-lapse video and resulting in an obvious difference in neutrophil movement between *kras+* and *kras−* larvae in the presence of doxycycline. In a 1-hour time-lapse video of 8 dpf *kras+* larvae (Supplementary Video 1), neutrophils had active migratory movement surrounding the liver. However, within the liver, infiltrated neutrophils were rather stationary with only minimal movement. In *kras−* siblings (Supplementary Video 2), in contrast, neutrophils were uniformly active both within and outside the liver. The difference was further illustrated by tracking neutrophil movement from these videos using the Imaris software (Fig. 4A and B). Thus, the tumor microenvironment leads to an inactive migratory behavior of infiltrated neutrophils.

### Increase of segmented nuclei of TANs

It has been reported that TANs in rodent models have distinct nuclear morphology with a high percentage of segmented nuclei [4]. To examine whether our isolated TANs also displayed similar nuclear morphology, Giemsa staining on FACS-isolated neutrophils was performed. The neutrophil nuclei were characterized into three broad categories according to Fridlender *et al.* [4]: i) round nuclei denoting a naive status; ii) bi-lobed structure representing fully differentiated and mature neutrophils; iii) hyper-segmented nuclei frequently associated with tumors (Fig. 4C). In both *kras−* and *kras+* transgenic zebrafish, round nuclei represented 20-30% of the isolated neutrophils. In *kras−* larvae, the remaining neutrophils had bi-lobed nuclei (75%) and only a very small percentage (3%) had hyper-segmented nuclei (Fig. 4D). However, in *kras+* larvae, hyper-segmented nuclei neutrophils made up of 43% of total neutrophil population (Fig. 4D), suggesting that our isolated neutrophils from the doxycycline-induced *kras+* larvae were indeed enriched with TANs.

### Promotion of proliferation and suppression of apoptosis in kras^G12V^-expressing hepatocytes by infiltrated neutrophils

**Fig. 5:**
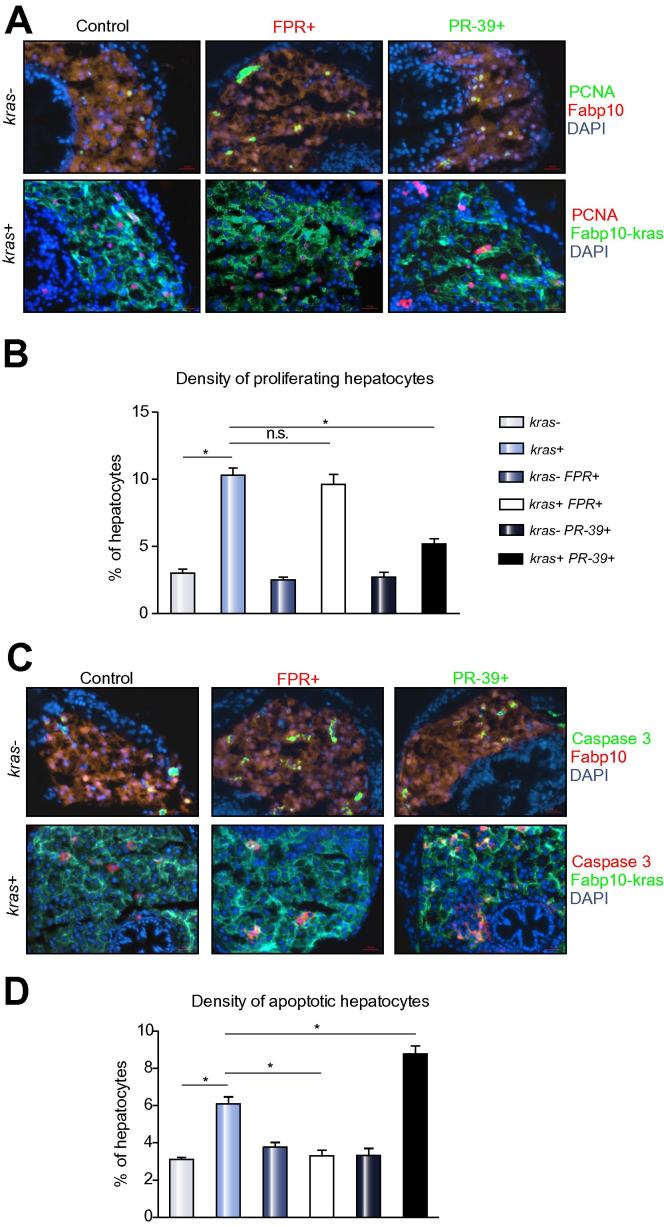
**Proliferation and apoptosis analyses of effects of neutrophils on oncogenic hepatocytes.** 8 dpf *fabp10+* and *kras+* larvae after exposure to FPR A14 or PR-39 with doxycycline were analysed by immunohistochemistry. (A and C) PCNA (A) or active caspase 3 (B) staining with representative images from each group. Control *Fabp10+* larvae with DsRed expression in hepatocytes were stained with Alexa Fluor 488-conjugated secondary antibody after the primary antibody incubation (top rows) while *kras+* larvae with GFP expression in hepatocytes were stained with Alexa Fluor 568-conjugated secondary antibody (bottom rows). All sections were counter-stained with DAPI. (B and D) Quantification of proliferating (B) and apoptotic (D) hepatocytes as percentage of total hepatocytes (n = 10; ^*^p <0.05; n.s., no significance).

To identify the causative factors for liver size change when neutrophil activity was modulated, hepatocyte proliferation and apoptosis were examined. As shown in Fig. 5, there were significant increases of hepatocyte proliferation and apoptosis in doxycycline-induced *kras+* larvae, suggesting highly aberrant cell division and death in oncogenic livers. While co-exposure to FPR-A14/doxycycline did not induce a further increase of proliferating hepatocytes, it did lead to a significant reduction of apoptotic hepatocytes. In contrast, co-treatment of *kras+* larvae with PR-39/doxycycline reduced the number of proliferating hepatocytes and increased the number of apoptotic hepatocytes. Thus, the changes in neutrophil activity affect both proliferation and apoptosis of hepatocytes.

### Moderated histological phenotype of oncogenic hepatocytes by inhibition of neutrophil activity

**Fig. 6:**
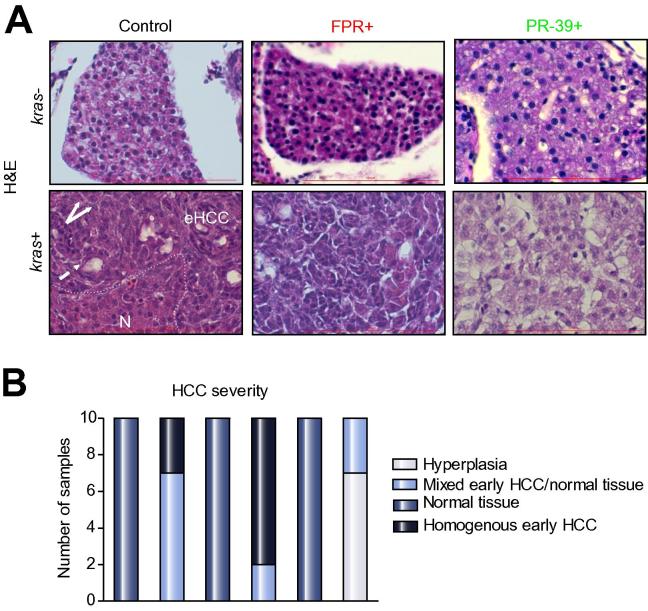
**Histological analyses of effects of neutrophils on hepatocarcinogenesis.** (A) H&E staining of liver sections. 8 dpf *kras−/lyz+* and *kras+/lyz+* larvae after exposure to FPR A14 or PR-39 with or without doxycycline, as indicated in the pictures, and were subject to H&E staining. Abbreviations: eHCC, early HCC; N, normal liver region. Dashed arrow: pseudo glandular pattern; white arrows: enlarged nuclei. (B) Quantification of phenotype observed in liver histology sections (n = 10, ^*^p <0.05).

Histologically, as shown in Fig. 6A, all H&E staining of 8 dpf *kras+* larvae exposed to doxycycline showed histological features of early carcinoma, e.g. disorganized cell plate, large nucleus-to-cytoplasm ratio and pseudo glandular patterns. All *kras+* larvae exposed to FPR-A14 and doxycycline showed similar early carcinoma histology, but with significantly less proportion of normal tissue, suggesting that the additional exposure to FPR-A14 had further stimulated HCC progression. In contrast, most *kras+* larvae exposed to PR-39 and doxycycline did not display the early carcinoma phenotype and instead resembles closely to the liver phenotype of control *kras−* larvae, implying exposure to PR-39 deters progression into HCC. Fig. 6B summarizes the quantitative distribution of histological phenotypes in each treatment group, further indicating that inhibition of neutrophils led to moderated tumor phenotype while activation of neutrophils deteriorated the phenotype. The liver phenotype was also examined by immunostaining using two fibrosis markers, Laminin and Collagen 1a [30] (Supplementary Fig. 2) and we observed increases of both biomarkers in induced *kras+* oncogenic livers, which were further increased by FPR-A14 but attenuated by PR-39, consistent with the general trends of liver tumor severity based on H&E staining.

### Pro-inflammatory microenvironment of oncogenic liver and reduction of anti-tumor activities of TANs

**Fig. 7:**
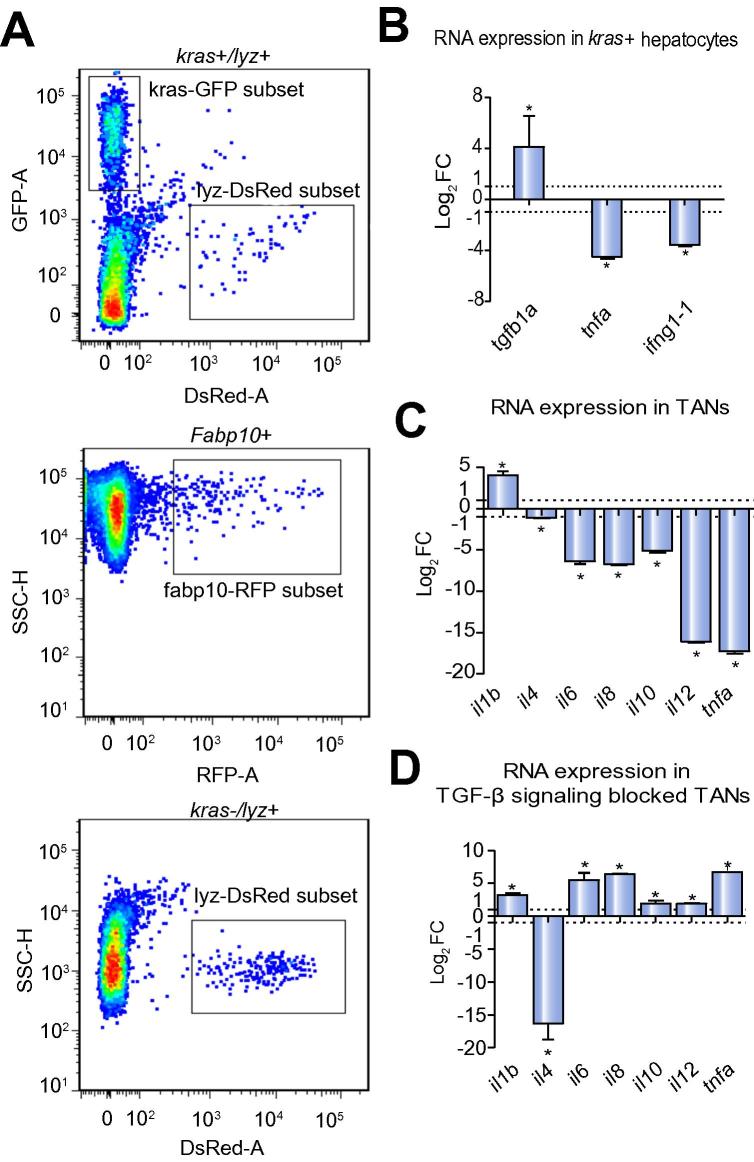
**Analyses of FACS-isolated hepatocytes and neutrophils.** (A) FACS profiles of liver cells from *kras+/lyz+*, *fabp10+* and *kras−/lyz+* fish. Fluorescent protein-labeled hepatocytes and neutrophils are boxed in each profile. Both cell populations are boxed. (B–D) RT-qPCR determination of RNA expression of selected genes in *kras^V12^*-expressing hepatocytes (B), TANs (C) and Tgf-β signaling blocked TANs (D). Fold changes in log2 scale are shown for *kras^V12^*-expressing hepatocytes *vs*. *kras^V12^* non-expressing *fabp10+* hepatocytes (B), TANs *vs*. NNs (C) and Tgf-β signaling blocked TANs *vs*. control TANs (D). All quantifications were repeated in three experiments and the same trend was observed. ^*^p <0.05. (This figure appears in colour on the web.)

It has been well established that cancer cells are capable of creating a pro-inflammatory microenvironment [31], [32]. To investigate molecular interaction of oncogenic hepatocytes and neutrophils in our model, GFP*-kras^G12V^*-expressing hepatocytes were isolated from doxycycline-treated *kras+* larvae by FACS and meanwhile enriched TANs were isolated based on DsRed expression (Fig. 7A). RT-qPCR analysis of *kras+* hepatocytes (Fig. 7B) showed a significant upregulation of *tgfβ1a*, a primary regulator in early development of liver fibrosis and cancer-related inflammation [33], [34]. In contrast, key anti-tumor genes such as *tnfa* and *ifnγ* showed significant downregulation. Meanwhile, compared to NNs, TANs showed a general pro-tumor gene expression pattern. For instance, *il1b*, which promotes early cancer angiogenesis [35], was significantly upregulated while anti-tumor cytokines, *il4*, *il6*, *il8*, *il10*, *il12*, and *tnfa*, all showed significant downregulation (Fig. 7C). Tgf-β is known to polarize neutrophils to a pro-tumor phenotype [4]. To investigate if oncogenic hepatocyte-secreted Tgf-β mediated crosstalk between the two cell types, SB431542, a specific inhibitor of Tgf-β type I receptor [36], [37], was used to block Tgf-β signaling in TANs where both *tgfβr1a* and *tgfβr1b* were found to be expressed (data not shown). Compared to unblocked TANs, significant upregulation of all but one examined anti-tumor cytokines (*il6*, *il8*, *il10*, *il12*, and *tnfa*) was observed in Tgf-β receptor blocked TANs. Thus, blockage of Tgf-β signaling partially rescued the expression of these anti-tumor genes (Fig. 7D).

**Fig. 8:**
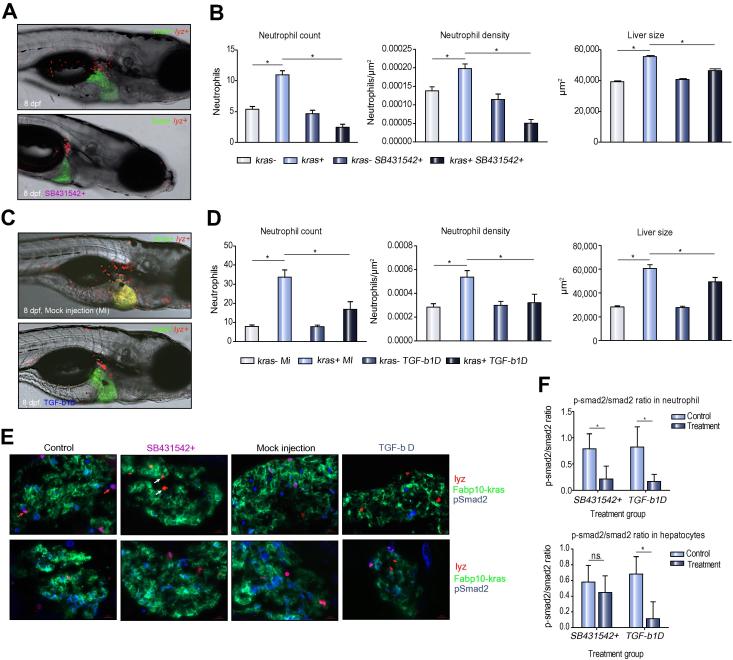
**Effect of antibody-mediated Tgf-β depletion on TAN recruitment.** (A) Representative images of 8 dpf *kras+/lyz+* larvae exposed to either doxycycline alone (top) or with SB431542 (bottom). (B) Neutrophil counts (left) and density (middle) in the liver and liver size (right) after SB431542 treatment. (C) Representative images of 8 dpf *kras+/lyz+* larvae injected with Tgf-β antibody (bottom) or mock injected as a control (top). Both were induced by doxycycline. (D) Neutrophil counts (left) and density (middle) in the liver and liver size (right) in Tgf-β-depleted (tgfb-1D) and mock injected (MI) larvae. (E) Validation of SB431542 exposure and Tgf-β depletion by immunostaining of Smad2 and phospho-Smad2 (pSmad2). Representative liver sections are shown from each group as indicated. The color code of each probes correspond the color signals in the images. Red arrows, DsRed expressing neutrophils with pSmad2 expression; white arrows, DsRed expressing neutrophils without pSmad2 expression. (F) Ratios of pSmad2/smad2 in neutrophils (top) and hepatocytes (bottom) in SB431542-inhibited and Tgf-β depleted larvae. n = 10; ^*^p <0.05.

Since Tgf-β has been reported to be a chemoattractant for neutrophils [38], we further depleted Tgf-β by injection of zebrafish Tgf-β antibody into 3 dpf *kras+/lyz+* zebrafish as well as by SB431542 inhibition of TGF-β receptor [39]. We noticed reduced neutrophils in the liver by 8 dpf by both approaches (Fig. 8A and C) as confirmed by the decreases in neutrophil counts and density in the liver, which was also accompanied with a decrease in liver size (Fig. 8B and D). The successful depletion of Tgf-β was supported by significant reductions of phosphorylated Smad2, a downstream marker of the Tgf-β pathway in both neutrophils and hepatocytes (Fig. 8E–F). Collectively, these experiments demonstrate the role of Tgf-β in attracting neutrophils to the liver upon oncogenic *kras* activation in hepatocytes.

## Discussion

### Rapid response of neutrophils to a pro-inflammatory microenvironment created by oncogenic krasV12-expressing hepatocytes

The chronic, unresolved inflammation is well recognized as one of the hallmarks and a contributing factor of early HCC. Persistent inflammation of the liver drives hepatocyte apoptosis and compensatory proliferation, while prolonged abnormal regeneration potentially promotes hepatic damage, fibrosis, cirrhosis and eventually HCC [40]. TGF-β plays a crucial role in the progression of the liver disease as the initial damage of liver upregulates TGF-β to lead to a wound healing response [41]. In our *kras^V12^* transgenic model, *kras^G12V^*-expressing hepatocytes displayed an upregulation of *tgfβ1a* and downregulation of anti-tumor genes, *tnfa* and *ifnγ*. The gene expression pattern indicated that the oncogenic *kras+* hepatocytes favored a pro-inflammatory environment, consistent with a previous report of liver tumors arising from inflammation due to chronic injury [42]. Moreover, decreased expression of *tnfa* and *ifnγ* aids in the creation of a pro-tumor microenvironment, which is in line with clinical HCC data [43], [44]. Thus, oncogenic *kras^V12^* expression have created a pro-inflammatory microenvironment in the HCC initiation stage of HCC and downregulation of important anti-tumor genes further facilitates the disease progression. Moreover, TAN showed significant downregulation of a variety of anti-tumor cytokines, including *il4*, *il6*, *il8*, *il10*, *il12*, and *tnfa*, and upregulation of pro-tumor *il1b*. These observations indicate a crosstalk between the oncogenic hepatocytes and neutrophils. Oncogenic hepatocytes secrete high level of Tgf-β which recruit neutrophils as it has been demonstrated to be a potent chemoattractant of neutrophils [38]. We further validated that depletion of Tgf-β resulted in a significant reduction of neutrophil density in the oncogenic livers, which caused a decrease of oncogenic liver size compared to oncogenic liver without Tgf-β depletion (Fig. 8D). However, the liver size remained significantly larger than the size of livers in *kras−* controls, which may indicate multiple roles of Tgf-β, including a suppressor in early tumor progression [45]. Once the neutrophils are recruited, a high level of Tgf-β in oncogenic hepatocytes further causes TANs to reduce expression of anti-tumor genes and increase in *il1b.* Increased expression of *il1b* in TANs hints the possible angiogenesis promoting role of TAN in HCC. Blockade of the Tgf-β signaling pathway by SB431542 led to a partial rescue of the pro-tumor gene expression profile in TAN, further validating the molecular crosstalk between the two cell types.

### Acceleration of HCC progression by TANs

Our data indicated a good correlation between the number/density of liver-infiltrated neutrophils and the size of oncogenic liver; thus, it is likely that neutrophils play a stimulating role in liver tumor initiation and progression. This phenomenon has been further confirmed by pharmacological experiments. Both the general immune stimulator LPS and neutrophil-specific activator FRP-A14 caused further increase of liver-infiltrated neutrophils and also further increase of the liver size. Conversely, neutrophil inhibitor, PR-39, caused a reduction of liver-infiltrated neutrophils and also slowed down the growth of oncogenic liver. The observed effects by FRP-A14 and PR-39 appear to be mainly due to the change of neutrophil numbers in the liver as neither chemical affects the number of macrophages in the liver (Supplementary Fig. 3). In addition, using Irf8 morpholino to deplete macrophages and thus increase neutrophil density, we demonstrated that neutrophil alone also have a significant effect in accelerating HCC progression.

Histologically, the severity of oncogenic livers also correlated to the activity of neutrophils. There was a rapid oncogenic transformation of hepatocytes upon *kras^G12V^* induction and histological HCC phenotype could be observed within four days of doxycycline induction. Neutrophils likely play an important role in this process as inhibition of neutrophils by PR-39 resulted in a liver histology close to a normal liver. The possible explanation of the neutrophil-accelerated tumorigenesis lies in the ability of neutrophils to induce proliferation and inhibit apoptosis in oncogenic hepatocytes. Enhancement of the neutrophil activity using an activator showed no further increase in hepatocyte proliferation but significant inhibition of apoptosis. In contrast, inhibition of neutrophil activity with PR-39 led to inhibition of proliferation and promotion of apoptosis in hepatocytes (Fig. 5B and D).

### Characteristic and behavior changes of neutrophils in tumor microenvironment

It has been well documented that neutrophils are a heterogeneous population and the characteristic and behavior of TANs and non-infiltrated neutrophils are markedly different [4]. In our study, we also noticed several major differences between TANs from oncogenic *kras+* larvae and NNs from *kras*− control larvae. First, migratory patterns of TANs appear to be relatively active when the cells were meandering along the tumor perimeter, and become relative motionless in the tumor, while the migration of NNs in *kras−* larvae are uniformly active regardless of their locations. Second, Giemsa staining of TANs showed a high percentage of hyper-segmented nuclei, which is consistent with the report that pro-tumor N2 neutrophils in mouse models have hyper-segmented nuclear structure while anti-tumor N1 neutrophils have largely round nucleus morphology [4]. Third, by RT-qPCR analyses, TANs showed downregulation of anti-tumor genes (e.g., *il4*, *il6*, *il8*, *il10*, *il12*, and *tnfa*) and upregulation of pro-tumor genes such as *il1b*, which also promotes early cancer angiogenesis [35], indicating a potential role of TANs in pro-angiogenesis in HCC initiation. A complete search for more critical genes expressed in TANs for promoting early hepatocarcinogenesis could be carried out in the future by RNA-Seq analyses in our present model.

In summary, our study suggests that oncogenic *kras^G12V^* expression in hepatocytes favors a pro-inflammatory microenvironment by increased Tgf-β1a expression, which attracts a rapid recruitment of neutrophils to oncogenic livers. These liver-infiltrated neutrophils have stimulating roles in early hepatocarcinogenesis and they lost anti-tumor activity. Our data are consistent with the view that the presence of neutrophils in the tumor microenvironment is an important marker in the aggressiveness of the liver cancer progression [46], [47]. A high neutrophil density in the tumor would be an indicator of high hyperplasia and low apoptosis. Thus, Neutrophils are not bystanders in hepatocarcinogenesis and instead they are actively promoting its initiation and progression.

## Financial support

This work was supported by a grant from National Medical Research Council, Singapore (R154000473272). Y.F. is funded by a Wellcome Trust Sir Henry Dale Fellowship (100104/Z/12/Z).

## Conflict of interest

The authors who have taken part in this study declared that they do not have anything to disclose regarding funding or conflict of interest with respect to this manuscript.

## Authors’ contributions

Conceived and designed the experiments: CY, XH, SW, YF, ZG. Performed the experiments: CY, XH. Analysed the data: CY, XH, ZG. Contributed reagents/materials/analysis tools: SW, YF. Wrote the paper: CY, XH, ZG.

## References

[1] Piccard H., Muschel R.J., Opdenakker G. (2012). On the dual roles and polarized phenotypes of neutrophils in tumor development and progression. Crit Rev Oncol Hematol.

[2] Zivkovic M., Poljak-Blazi M., Zarkovic K., Mihaljevic D., Schaur R.J., Zarkovic N. (2007). Oxidative burst of neutrophils against melanoma B16–F10. Cancer Lett.

[3] di Carlo E., Iezzi M., Pannellini T., Zaccardi F., Modesti A., Forni G. (2001). Neutrophils in anti-cancer immunological strategies: old players in new games. J Hematother Stem Cell Res.

[4] Fridlender Z.G., Sun J., Kim S., Kapoor V., Cheng G., Ling L. (2009). Polarization of tumor-associated neutrophil phenotype by TGF-beta: “N1” versus “N2” TAN. Cancer Cell.

[5] Zhao J.J., Pan K., Wang W., Chen J.G., Wu Y.H., Lv L. (2012). The prognostic value of tumor-infiltrating neutrophils in gastric adenocarcinoma after resection. PLoS One.

[6] Shang K., Bai Y.P., Wang C., Wang Z., Gu H.Y., Du X. (2012). Crucial involvement of tumor-associated neutrophils in the regulation of chronic colitis-associated carcinogenesis in mice. PLoS One.

[7] Opdenakker G., Van Damme J. (1992). Cytokines and proteases in invasive processes: molecular similarities between inflammation and cancer. Cytokine.

[8] Jablonska J., Leschner S., Westphal K., Lienenklaus S., Weiss S. (2010). Neutrophils responsive to endogenous IFN-beta regulate tumor angiogenesis and growth in a mouse tumor model. J Clin Invest.

[9] Coussens L.M., Raymond W.W., Bergers G., Laig-Webster M., Behrendtsen O., Werb Z. (1999). Inflammatory mast cells up-regulate angiogenesis during squamous epithelial carcinogenesis. Genes Dev.

[10] Feng Y., Santoriello C., Mione M., Hurlstone A., Martin P. (2010). Live imaging of innate immune cell sensing of transformed cells in zebrafish larvae: parallels between tumor initiation and wound inflammation. PLoS Biol.

[11] Freisinger C.M., Huttenlocher A. (2014). Live imaging and gene expression analysis in zebrafish identifies a link between neutrophils and epithelial to mesenchymal transition. PLoS One.

[12] Kuang D.M., Zhao Q., Wu Y., Peng C., Wang J., Xu Z. (2011). Peritumoral neutrophils link inflammatory response to disease progression by fostering angiogenesis in hepatocellular carcinoma. J Hepatol.

[13] Alison M.R., Nicholson L.J., Lin W.R. (2011). Chronic inflammation and hepatocellular carcinoma. Recent Results Cancer Res.

[14] Greten T.F., Papendorf F., Bleck J.S., Kirchhoff T., Wohlberedt T., Kubicka S. (2005). Survival rate in patients with hepatocellular carcinoma: a retrospective analysis of 389 patients. Br J Cancer.

[15] Thomas M.B., O’Beirne J.P., Furuse J., Chan A.T., Abou-Alfa G., Johnson P. (2008). Systemic therapy for hepatocellular carcinoma: cytotoxic chemotherapy, targeted therapy and immunotherapy. Ann Surg Oncol.

[16] El-Serag H.B., Marrero J.A., Rudolph L., Reddy K.R. (2008). Diagnosis and treatment of hepatocellular carcinoma. Gastroenterology.

[17] Llovet J.M., Ricci S., Mazzaferro V., Hilgard P., Gane E., Blanc J.F. (2008). Sorafenib in advanced hepatocellular carcinoma. N Engl J Med.

[18] Chew T.W., Liu X.J., Liu L., Spitsbergen J.M., Gong Z., Low B.C. (2013). Crosstalk of Ras and Rho: activation of RhoA abates Kras-induced liver tumorigenesis in transgenic zebrafish models. Oncogene.

[19] Li Z., Huang X., Zhan H., Zeng Z., Li C., Spitsbergen J.M. (2012). Inducible and repressable oncogene-addicted hepatocellular carcinoma in Tet-on xmrk transgenic zebrafish. J Hepatol.

[20] Li Z., Zheng W., Wang Z., Zeng Z., Zhan H., Li C. (2013). A transgenic zebrafish liver tumor model with inducible Myc expression reveals conserved Myc signatures with mammalian liver tumors. Dis Model Mech.

[21] Nguyen A.T., Emelyanov A., Koh C.H., Spitsbergen J.M., Parinov S., Gong Z. (2012). An inducible kras(V12) transgenic zebrafish model for liver tumorigenesis and chemical drug screening. Dis Model Mech.

[22] Chew T.W., Liu X.J., Liu L., Spitsbergen J.M., Gong Z., Low B.C. (2014). Crosstalk of Ras and Rho: activation of RhoA abates Kras-induced liver tumorigenesis in transgenic zebrafish models. Oncogene.

[23] Hall C., Flores M., Storm T., Crosier K., Crosier P. (2007). The zebrafish lysozyme C promoter drives myeloid-specific expression in transgenic fish. BMC Dev Biol.

[24] Korzh S., Pan X., Garcia-Lecea M., Winata C.L., Wohland T., Korzh V. (2008). Requirement of vasculogenesis and blood circulation in late stages of liver growth in zebrafish. BMC Dev Biol.

[25] Ellett F., Pase L., Hayman J.W., Andrianopoulos A., Lieschke G.J. (2011). Mpeg1 promoter transgenes direct macrophage-lineage expression in zebrafish. Blood.

[26] Novoa B., Bowman T.V., Zon L., Figueras A. (2009). LPS response and tolerance in the zebrafish (Danio rerio). Fish Shellfish Immunol.

[27] Schepetkin I.A., Kirpotina L.N., Khlebnikov A.I., Quinn M.T. (2007). High-throughput screening for small-molecule activators of neutrophils: identification of novel N-formyl peptide receptor agonists. Mol Pharmacol.

[28] Hoffmeyer M.R., Scalia R., Ross C.R., Jones S.P., Lefer D.J. (2000). PR-39, a potent neutrophil inhibitor, attenuates myocardial ischemia-reperfusion injury in mice. Am J Physiol Heart Circ Physiol.

[29] Liongue C., Hall C.J., O’Connell B.A., Crosier P., Ward A.C. (2009). Zebrafish granulocyte colony-stimulating factor receptor signaling promotes myelopoiesis and myeloid cell migration. Blood.

[30] Huang M., Chang A., Choi M., Zhou D., Anania F.A., Shin C.H. (2014). Antagonistic interaction between Wnt and Notch activity modulates the regenerative capacity of a zebrafish fibrotic liver model. Hepatology.

[31] Grivennikov S.I., Greten F.R., Karin M. (2010). Immunity, inflammation, and cancer. Cell.

[32] Kuraishy A., Karin M., Grivennikov S.I. (2011). Tumor promotion via injury- and death-induced inflammation. Immunity.

[33] Matsuzaki K., Murata M., Yoshida K., Sekimoto G., Uemura Y., Sakaida N. (2007). Chronic inflammation associated with hepatitis C virus infection perturbs hepatic transforming growth factor beta signaling, promoting cirrhosis and hepatocellular carcinoma. Hepatology.

[34] Yang L., Inokuchi S., Roh Y.S., Song J., Loomba R., Park E.J. (2013). Transforming growth factor–β signaling in hepatocytes promotes hepatic fibrosis and carcinogenesis in mice with hepatocyte-specific deletion of TAK1. Gastroenterology.

[35] Carmi Y., Dotan S., Rider P., Kaplanov I., White M.R., Baron R. (2013). The role of IL-1beta in the early tumor cell-induced angiogenic response. J Immunol.

[36] Sun Z., Jin P., Tian T., Gu Y., Chen Y.G., Meng A. (2006). Activation and roles of ALK4/ALK7-mediated maternal TGFbeta signals in zebrafish embryo. Biochem Biophys Res Commun.

[37] Halder S.K., Beauchamp R.D., Datta P.K. (2005). A specific inhibitor of TGF-β receptor kinase, SB-431542, as a potent antitumor agent for human cancers. Neoplasia.

[38] Reibman J., Meixler S., Lee T.C., Gold L.I., Cronstein B.N., Haines K.A. (1991). Transforming growth factor beta 1, a potent chemoattractant for human neutrophils, bypasses classic signal-transduction pathways. Proc Natl Acad Sci U S A.

[39] Inman G.J., Nicolas F.J., Callahan J.F., Harling J.D., Gaster L.M., Reith A.D. (2002). SB-431542 is a potent and specific inhibitor of transforming growth factor-beta superfamily type I activin receptor-like kinase (ALK) receptors ALK4, ALK5, and ALK7. Mol Pharmacol.

[40] Weber A., Boege Y., Reisinger F., Heikenwalder M. (2011). Chronic liver inflammation and hepatocellular carcinoma: persistence matters. Swiss Med Wkly.

[41] Margadant C., Sonnenberg A. (2010). Integrin-TGF-beta crosstalk in fibrosis, cancer and wound healing. EMBO Rep.

[42] Khatib A.M., Auguste P., Fallavollita L., Wang N., Samani A., Kontogiannea M. (2005). Characterization of the host proinflammatory response to tumor cells during the initial stages of liver metastasis. Am J Pathol.

[43] Lee I.C., Huang Y.H., Chau G.Y., Huo T.I., Su C.W., Wu J.C. (2013). Serum interferon gamma level predicts recurrence in hepatocellular carcinoma patients after curative treatments. Int J Cancer.

[44] Kuppen P.J., Jonges L.E., van de, Velde C.J., Vahrmeijer A.L., Tollenaar R.A., Borel Rinkes I.H. (1997). Liver and tumour tissue concentrations of TNF-alpha in cancer patients treated with TNF-alpha and melphalan by isolated liver perfusion. Br J Cancer.

[45] Tang B., Vu M., Booker T., Santner S.J., Miller F.R., Anver M.R. (2003). TGF-beta switches from tumor suppressor to prometastatic factor in a model of breast cancer progression. J Clin Invest.

[46] Rao H.-L., Chen J.-W., Li M., Xiao Y.-B., Fu J., Zeng Y.-X. (2012). Increased intratumoral neutrophil in colorectal carcinomas correlates closely with malignant phenotype and predicts patients’ adverse prognosis. PLoS One.

[47] Wang J., Jia Y., Wang N., Zhang X., Tan B., Zhang G. (2014). The clinical significance of tumor-infiltrating neutrophils and neutrophil-to-CD8+ lymphocyte ratio in patients with resectable esophageal squamous cell carcinoma. J Transl Med.

