# Supplementary material for: Stimulation of hepatocarcinogenesis by neutrophils upon induction of oncogenic kras expression in transgenic zebrafish
Source: J Hepatol. 2015 Aug;63(2):420–8. doi: 10.1016/j.jhep.2015.03.024 (PMC4508360; doi:10.1016/j.jhep.2015.03.024)
Supplement: Supplementary data [file mmc1.pdf]

Supplementary data to:

Stimulation of hepatocarcinogenesis by neutrophils upon induction of oncogenic *krasexpression in transgenic zebrafish*

Chuan Yan[1,2,§], Xiaojing Huo[1,§], Shu Wang[1], Yi Feng[3], Zhiyuan Gong[1,2,*]

Table of Content

Supplementary Materials and Methods

Supplementary Table 1

Supplementary Fig. 1

Supplementary Fig. 2

Supplementary Fig. 3

Supplementary Fig. 4

Supplementary Fig. 5

Supplementary Materials and Methods

Morpholino knock-down and Tgf-β depletion

For knockdown of Gcsfr and Irf8, previously validated morpholinos targeting each gene's splice-site respectively, MO-gcsfr (5′-GAAGCACAAGCGAGACGGATGCCAT-3′) (1) and MO-irf8 (5'-AATGTTTCGCTTACTTTGAAAATGG-3') (2), were used. A standard control morpholino, MO-SC, (5′-CCTCTTACCTCAGTTACAATTTATA-3′) targeting a human beta-globin intron (Gene Tools, Philomath, OR), was used as a negative control. Aliquots of morpholino (1 mM) and 1% (wt/vol) phenol red in Danieau solution were injected into embryos at the 1-cell stage. Doxycycline was added to all larvae from 3 dpf to 6 dpf. For Tgf-β depletion, antibody against zebrafish Tgf-β1 (Anaspec, 55450) was injected into 3-dpf *kras*+ larvae (1:100 dilution) at the anterior end of yolk.

Photography and image analysis

At each time point of chemical treatment, morpholino knockdown and antibody depletion experiments, 15-20 larvae of each group were randomly chosen for imaging. The larvae were anesthetized in 0.08% tricaine (Sigma, E10521) and immobilized in 3% methylcellulose (Sigma, M0521). Each larva was photographed separately using a confocal microscope (Carl Zeiss LSM510). 2D measurement of liver sizes was performed using ImageJ as previously described (3, 4) and neutrophils counted manually. Neutrophils in the trunk region from somites 1-4 were also counted as controls and we found no significant variations among all experimental conditions in this project (Supplementary Fig. 4). Time-lapse confocal microscopy was conducted on 8-dpf larvae embedded in 3% low-melting-point agarose and the time-lapse videos

were used for neutrophil tracking with multi-particle tracking by Imaris version 7.2.3 (Bitplane Scientific Software).

Isolation of hepatocytes and neutrophils by FACS

To enrich the TANs and liver cells, central part of 8-dpf larvae (after removal of the head and tail regions) were used for FACS using a cell sorter (BD Aria, 643245). The liver-enriched central parts were dissociated into single cells using a 40-µm mesh (BD falcon, 352340) and enzymatically digested with 0.05% trypsin (Sigma, T1426), as previously described (5). GFP+ oncogenic hepatocytes and Ds-Red+ TANs were isolated from doxycycline-induced *kras+/lyz+* transgenic larvae, while non-oncogenic control hepatocytes and NNs were isolated respectively from *fabp10+* and *kras-/lyz+* control larvae based on Ds-Red expression.

RNA extraction, cDNA amplification and RT-qPCR (reverse transcription-quantitative PCR)

Total RNA was extracted using RNeasy mini kit (Qiagen, 74104). A total of 5 ng RNA was used as a template to synthesize and amplify cDNA using QuantiTect Whole Transcriptome Kit (Qiagen, 207043). Amplified cDNA was carried out for real-time quantitative PCR with LightCycler 480 SYBR Green I Master (Roche, 04707516001). Interested genes were amplified by 40 cycles (95°C, 20 seconds; 65°C, 15 seconds; 72°C, 30 seconds). The sequences of primers used are presented in Supplementary Table 1. *Kras-EGFP* expression under various experimental conditions was measured by RT-qPCR and there was no significant change of the expression observed (Supplementary Fig. 5).

Histological and cytological analyses

8-dpf larvae were fixed in 4% paraformaldehyde in phosphate buffered saline (Sigma, P6748) and paraffin-sectioned at 5 μm thickness using a microtome, followed by hematoxylin and eosin (H&E) staining or immunohistochemistry. For immunohistochemistry, the primary antibody was rabbit anti-PCNA (Santa Cruz, FL-261), anti-caspase 3(BD biosciences, C92-065), anti-SMAD2 (Invitrogen, 700048), anti-phosphosmad2 (Millipore, 04-953), anti-collagen 1 (Abcam, ab23730) and anti-laminin (Thermo, RB-082). Giemsa staining was performed on isolated neutrophils were fixed in 100% methanol and subsequently stained with Giemsa (Sigma, GS500).

References

1. Feng Y, Renshaw S, Martin P. Live Imaging of Tumor Initiation in Zebrafish Larvae Reveals a Trophic Role for Leukocyte-Derived PGE2. Current Biology 2012;22:1253-1259.
2. Li L, Jin H, Xu J, Shi Y, Wen Z. Irf8 regulates macrophage versus neutrophil fate during zebrafish primitive myelopoiesis. Blood 2011;117:1359-1369.
3. Huang X, Zhou L, Gong Z. Liver tumor models in transgenic zebrafish: an alternative in vivo approach to study hepatocarcinogenes. Future Oncol 2012;8:21-28.
4. Li Z, Zheng W, Wang Z, Zeng Z, Zhan H, Li C, Zhou L, et al. A transgenic zebrafish liver tumor model with inducible Myc expression reveals conserved Myc signatures with mammalian liver tumors. Dis Model Mech 2013;6:414-423.
5. Manoli M, Driever W. Fluorescence-activated cell sorting (FACS) of fluorescently tagged cells from zebrafish larvae for RNA isolation. Cold Spring Harb Protoc 2012;2012.

Supplementary Table 1. Sequences of RT-qPCR primers

Gene symbol	Forward primer sequence (5'->3')	Reverse primer sequence (5'->3')
tnfa	CGTCTGCTTCACGCTCCATA	GATGTGCAAAGACACCTGGC
ifng1-1	ACAGATTTCGACGGTCCAGG	CCAATCCTTTGCAAGCGTGT
tgf-β1a	AGCAGAATTGCGTCTTCGGA	TCAAATGAGAGCCAGCGGTT
il1b	GCCTGTGTGTTTGGGAATCT	TGATAAACCAACCGGGACAT
il4	TCTCTGCCAAGCAGGAATGG	TTCATTGTGCATTCCCCCGA
il6	ATGACGGCATTTGAAGGGGT	TCAGGACGCTGTAGATTCGC
il8	GCGACAGCGTGGATCTACAG	GGCATTCACACCCAAAGCG
il10	CACAACCCCAATCGACTCCA	GAGCAAATCAAGCTCCCCCA
il12	AGGGCTCTTCGTTTGACGAC	TGTCATGCGGTGGTGTAGTG
egfp-kras	CCGCCCTGAGCAAAGACCCC	AGCGGTGTCCTTTCAATGAG

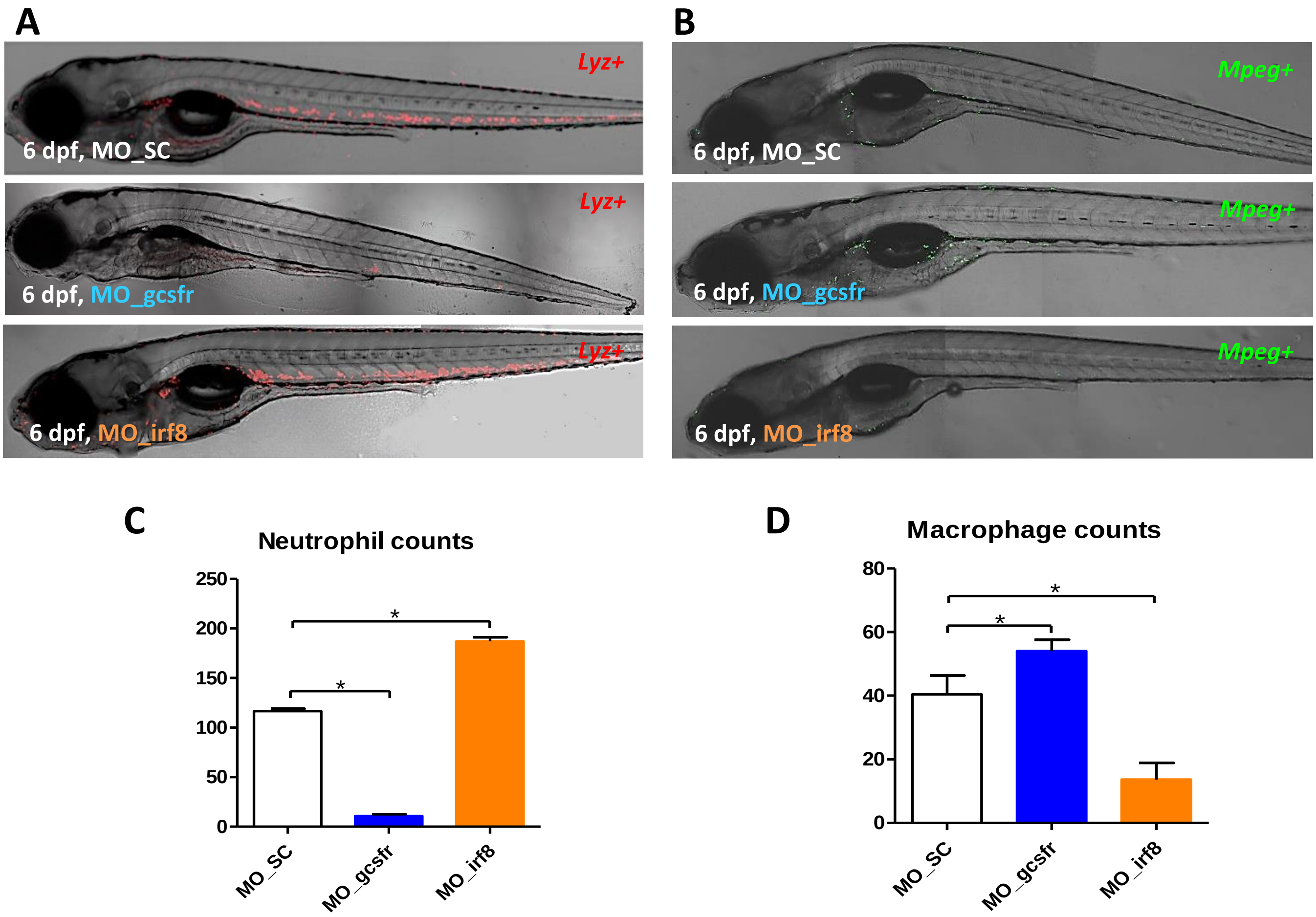

Supplementary Fig. 1. Effect of morpholino knockdown of Gcsfr and Irf8. (A) 6-dpf *lyz+* larvae injected with morpholinos MO-SC (top), MO-gcsfr (middle) or MO_irf8 (bottom). (B) 6-dpf *mpeg+* larvae injected with morpholinos MO-SC (top), MO-gcsfr (middle) or MO_irf8 (bottom). (C) Quantification of number of neutrophils per larva. (D) Quantification of number of microphages per larva. (n=5, *p<0.05).

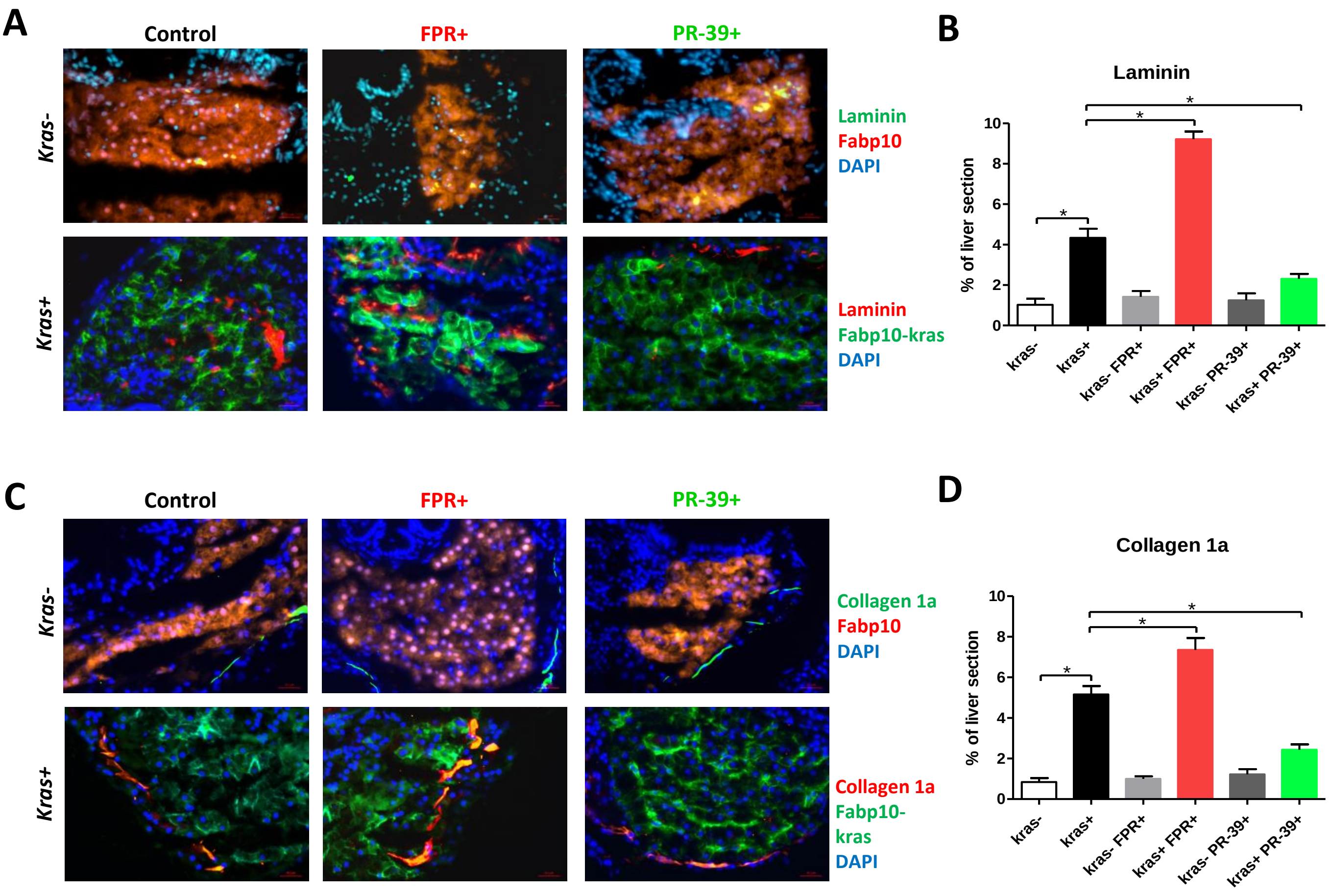

Supplementary Fig. 2. Effect of neutrophil infiltration on expression of Laminin and collagen I in the liver. 8-dpf *fabp10*+ and *kras*+ larvae after exposure to FPR-A14 or PR-39 with or without doxycycline were cryo-sectioned and immune-stained for Laminin and Collagen I expression. (A, B) Immuno-staining for Laminin (A) and quantification (B). (C, D) Immuno-staining for Collagen 1a (C) and quantification (D). Control *fabp10*+ larvae with DsRed expression in hepatocytes were stained with Alexa Fluor 488-conjugated secondary antibody after the primary antibody incubation (top rows) while *kras*+ larvae with GFP expression in hepatocytes were stained with Alexa Fluor 568-conjugated secondary antibody (bottom rows). All sections were counter-stained with DAPI. Treatment groups (n=10 each group) and color probes are indicated at the top and on the right respectively. Quantification of percentage of Laminin- or collagen I-positive areas out of total liver area is presented in (C) and (D). Statistical significance in quantification: *p<0.05.

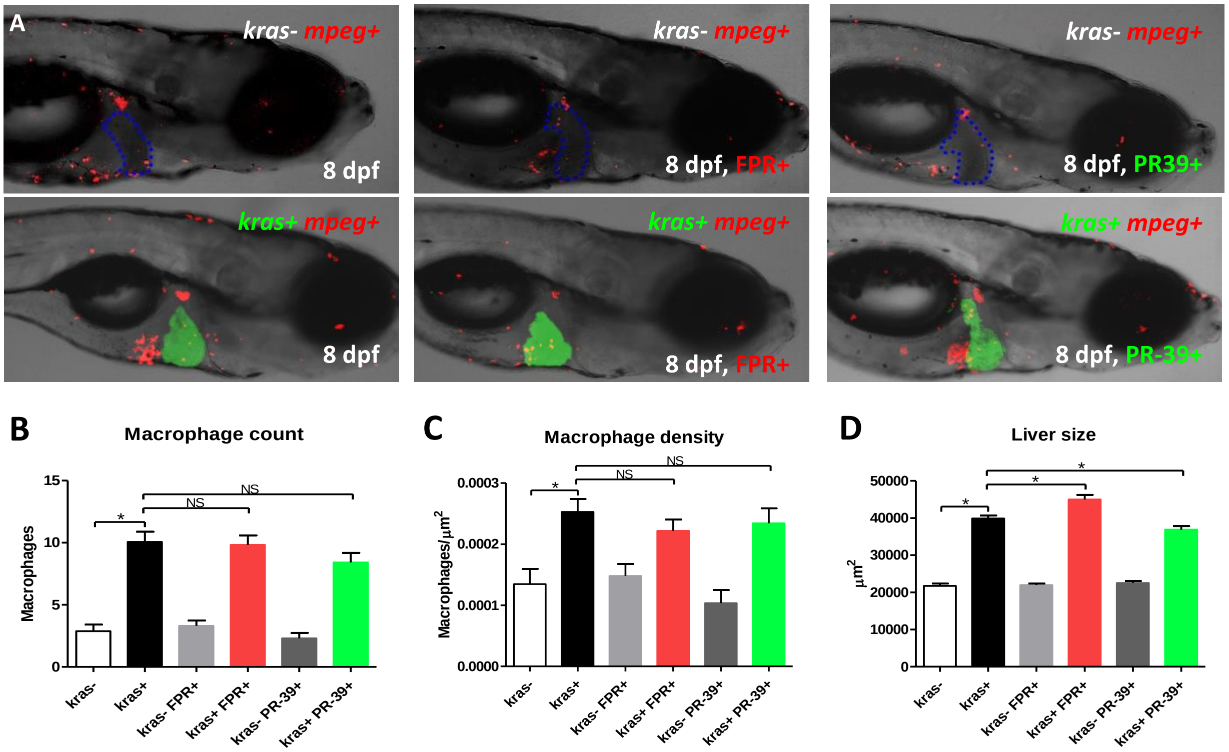

Supplementary Fig. 3. Lack of effect of FPRA14 and PR-39 on macrophages. (A) Representative images of 8-dpf *kras-/mpeg+* (top row) and *kras+/mpeg+* (bottom row) larvae treated with doxycycline alone (left), FPR-A14 and doxycycline (middle) or PR-39 and doxycycline (right) as indicated. Livers in *kras*- larvae are outlined. (B-D) Macrophage counts (B) and density (C) in the liver and liver size (D) after chemical treatment in the larvae (n>17 in each group). Statistical significant: *p<0.05.

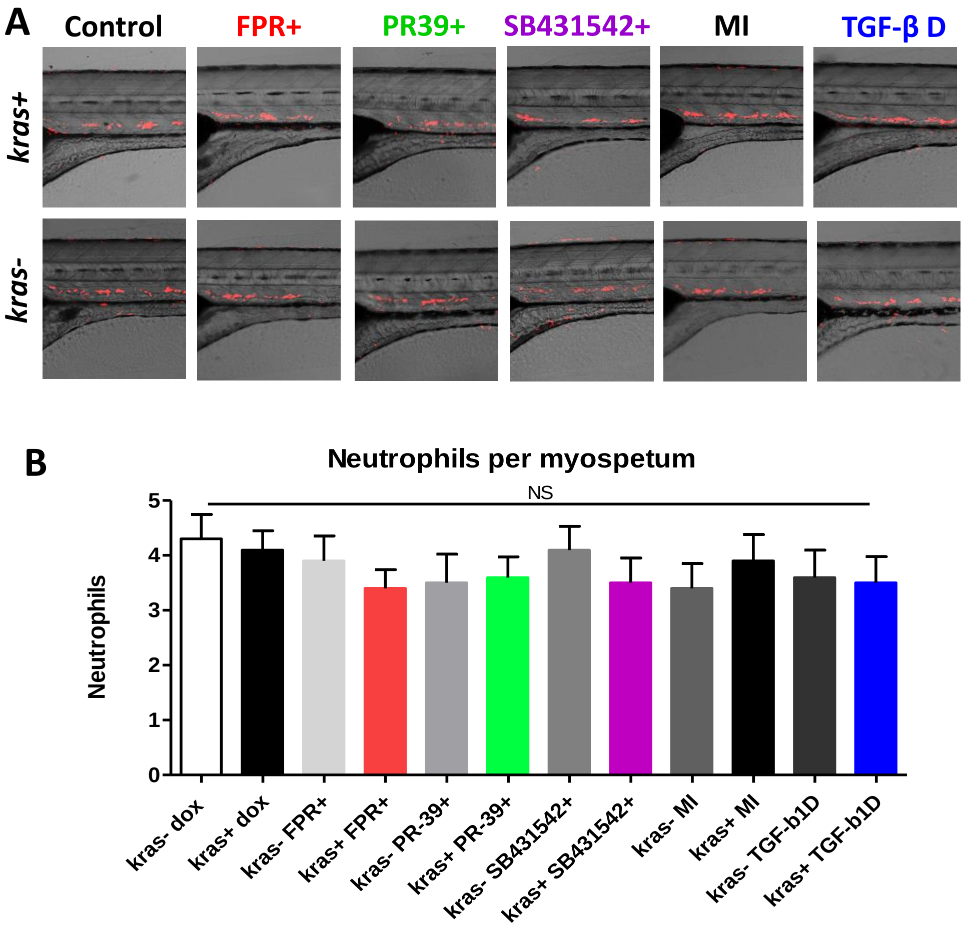

Supplementary Fig. 4. Lack of significant changes of neutrophil numbers under various experimental conditions. To support the specific changes of neutrophil numbers in the livers in various experimental conditions, the number of neutrophils were quantitated in the trunk region (somites 1-4). (A) Representative images of neutrophil distribution in the trunk of *kras*+ and *kras*- zebrafish larvae under various experimental conditions (from left to right), Control with doxycycline alone, FPR-A14 with doxycycline, PR39 with doxycycline, SB431542 with doxycycline, Mock injected, and Tgf-β depletion. (B) Quantification of numbers of neutrophils per somite among different treatment groups. (n=5 fish). NS, no significant difference.

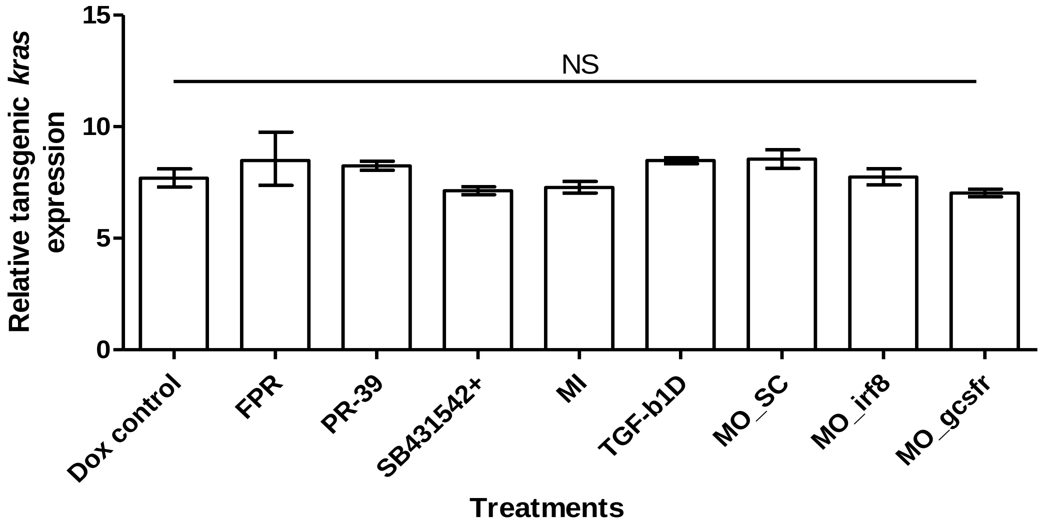

Supplementary Fig. 5. Lack of significant changes of transgenic *kras* expression under various experimental conditions. RT-qPCR quantification of *kras-egfp* RNA expression was carried out with total RNAs from 8-dpf *kras*+ larvae under various experimental conditions (from left to right): doxycycline alone, FPR-A14 with doxycycline, PR39 with doxycycline, SB431542 with doxycycline, Mock injected, Tgf-β depletion, and 6-dpf morphants after injection of MO_SC, MO_irf8 and MO_gcsfr. The level of *egfp-kras* expression was normalized with endogenous *β–actin* RNA expression. n=10 each group; NS, no significant difference.
